# A Practical Guide to Meat Inspection

**Published:** 1890-05

**Authors:** 


A Practical Guide to Meat Inspection. By Thomas Walley, M.R.C.V.S., Principal of the Edinburgh Royal (Dick's) Veterinary College. With 28 illustrations; pp. vi, 193. Edinburgh and London: Young J. Pentland, 1890.

This volume will meet, to a great extent, a long-felt want. While the work is, on the whole, very good, yet one cannot help thinking that it might be condensed at least one-half without detracting from its value. Altogether too much space is devoted to those diseases which are in no way directly transmissible from animal to man through the consumption of the meat.

The method recommended to be pursued in the examination of flesh for trichinæ, that of macerating small pieces of muscle in acetic acid before subjecting them to microscopic examination, is too slow for practical purposes and is unnecessary. The parasites are easily detected with a power of sixty diameters in small pieces of muscle crushed between two glass slides.

In the article on actinomykosis, while the author admits that the disease is due to a special fungus, and that, according to Crookshank, it has been transmitted from man to animals, he does not consider it justifiable to condemn the meat of such animals as unfit for food, because there is no direct evidence of its transmission from animals to man. The same argument would, it seems to us, apply to the meat of tuberculous animals, yet the author is very firm in his opinion that all such meat should be condemned.

As stated above, however, the work is, on the whole, very good, and will, no doubt, be found very useful to those engaged in the inspection of meat. A. W. C.

